# Comprehensive molecular docking and antifungal activity analysis of Moringa extracts targeting *Candida auris* dihydrofolate reductase (8CRH)

**DOI:** 10.1038/s41598-025-27930-w

**Published:** 2025-12-19

**Authors:** Maha S. I. Wizrah, Zeinab A. Yahia, Hanan Al-Omari, Norah D. Aldawsari, Ahlam Hamad Alrokban, Nada Ibrahim Alsugiran, Awais Khalid, Rasha Elsayim

**Affiliations:** 1 Department of Biology, College of Science and Humanities in Al-Kharj,, Prince Sattam Bin Abdulaziz University, Al-Kharj, 11942 Saudi Arabia; 2 Department of Biological Sciences, Faculty of Science, University of Jeddah, Jeddah, Saudi Arabia; 3 Department of Biology, College of Sciences, Princess Nourah bint Abdulrahman University, Riyadh, Saudi Arabia; 4 Department of Physics, College of Science and Humanities in Al-Kharj, Prince Sattam Bin Abdulaziz University, 11942 Al-Kharj, Saudi Arabia; 5 Department of Botany and Microbiology, College of Science, King Saud University, 11451 Riyadh, Saudi Arabia

**Keywords:** *Candida auris*, *Moringa*, DHFR inhibition, Natural antifungal, Molecular Docking, Biochemistry, Biotechnology, Drug discovery, Microbiology, Plant sciences

## Abstract

*Candida auris* is an emerging multidrug-resistant fungal pathogen posing serious global healthcare challenges due to its persistence, frequent misidentification, and resistance to all major antifungal drugs. This study aimed to explore natural therapeutic alternatives by investigating the antifungal and molecular properties of *Moringa peregrina* and *Moringa oleifera* leaf extracts. Ethanolic and aqueous extracts of both *Moringa species* were prepared and evaluated against *C. auris* and other *Candida species* (*Candida spp*.) using zone of inhibition (ZI), minimum inhibitory concentration (MIC), and minimum fungicidal concentration (MFC) assays. Phytochemical composition was characterized through fourier transform infrared spectroscopy (FTIR) and gas chromatography–mass spectrometry (GC–MS) analyses, while cytotoxicity on mammalian MCF-7 cells was assessed using the MTT assay. Molecular docking and 100-ns molecular dynamics simulations were conducted to examine the binding of key bioactive compounds to *C. auris* dihydrofolate reductase (DHFR; PDB: 8CRH). Ethanolic extracts exhibited the strongest antifungal activity (ZI: 16–16.5 mm; MIC: 0.5–0.7 mg/mL), whereas aqueous extracts were comparatively less effective. FTIR spectra revealed prominent peaks corresponding to hydroxyl (O–H), carbonyl (C = O), and C–O functional groups, confirming the presence of alcohols, esters, phenols, and carboxylic acids, while GC–MS identified β-sitosterol, stigmasterol, dihydroxanthin, and vitamin E derivatives as predominant constituents. Docking results revealed high binding affinities for vitamin E (− 86.6 kcal/mol) and stigmasterol (− 83.5 kcal/mol), exceeding that of fluconazole (− 27.3 kcal/mol). Molecular dynamics confirmed stable protein–ligand complexes, with hydrophobic and van der Waals forces dominating the interactions. Cytotoxicity assays revealed low toxicity at antifungal concentrations (IC₅₀ ≥ 120 µg/mL). These findings demonstrate that *Moringa* extracts—particularly the ethanolic fractions—harbor potent bioactive compounds with promising antifungal activity against *C. auris*, highlighting their potential as novel, plant-derived therapeutic candidates to combat resistant fungal infections.

## Introduction

*C. auris* is an emerging multidrug-resistant fungal pathogen that has rapidly become a global health concern. Since its initial discovery in 2009, it has been reported on multiple continents, often causing hospital outbreaks with high mortality rates. A major challenge in treating *C. auris* is its resistance to all three main classes of antifungal drugs: azoles, echinocandins, and polyenes. Resistance results from mechanisms such as mutations in ERG11 and FKS1, overexpression of efflux pumps, and biofilm formation. This adaptability significantly limits treatment options and has led the World Health Organization and the U.S. Centers for Disease Control and Prevention (CDC) to classify *C. auris* as an urgent health threat. Traditional antifungals primarily target ergosterol biosynthesis or fungal cell wall synthesis, but these pathways are increasingly compromised by resistance. Therefore, exploring alternative strategies, including new fungal targets, is essential. One such target is dihydrofolate reductase (DHFR), a key enzyme in folate metabolism and DNA synthesis. Inhibiting DHFR could bypass common resistance mechanisms and offer a new therapeutic route against resistant fungi^1^. Natural products are still a valuable source of antimicrobial agents, providing chemical diversity and multitarget mechanisms. Among them, species of the *Moringa genus* (*Moringa peregrina* and *Moringa oleifera*) are well known for their pharmacological properties, including antibacterial, antioxidant, anti-inflammatory, and anticancer activities. Their leaves contain secondary metabolites such as sterols, flavonoids, saponins, and phenolic compounds, many of which are associated with antimicrobial activity^2–6^. However, their potential against multidrug-resistant fungi, especially *C. auris*, remains underexplored. In this study, we examined the antifungal effectiveness of ethanolic and aqueous extracts of *M. peregrina* and *M. oleifera* against *C. auris* and other *Candida Spp*. Using a combined approach of in vitro assays, phytochemical profiling (FTIR and GC–MS), and in silico analysis (molecular docking and molecular dynamics simulations), we assessed both the antifungal potential and the mechanism of action of the bioactive compounds. We hypothesized that phytoconstituents from *Moringa* extracts could inhibit *C. auris* DHFR and provide new leads for antifungal drug development^4–6^.

## Methodology

### Plant material and extraction

The leaves of *M. peregrina* were procured from Wadi Ad-Dawasir, Saudi Arabia, while *M. oleifera* leaves were sourced from a local market. Species authentication was conducted by Dr. Haider Abdelgadir at the Medicinal and Aromatic Plant Research Institute (MAPRI). The leaves underwent washing, shade-drying, and subsequent pulverization. Twenty grams of each powdered sample were subjected to extraction using 100 mL of either ethanol or distilled water, with agitation maintained for 72 h. The resulting filtrates were processed through Whatman No. 1 filter paper. Ethanol was evaporated under sterile conditions, whereas aqueous extracts were filtered using a 0.25 μm membrane and air-dried. The dried extracts were stored at room temperature until further use,^7–9^.

### Phytochemical characterization

The FTIR was used on both ethanol and aqueous extracts within the 400–4000 cm⁻^1^ range to identify functional groups. To determine the main components of all *Morina* extracts GC–MS was employed (Agilent 7890A/5975C system with a DB-5MS column). Extracts were filtered through a 22 μm membrane, and 1 μL aliquots were analyzed under standard conditions (helium flow rate of 1 mL/min, injector temperature of 280 °C, column temperature of 300 °C, ionization energy of 70 eV)^10^.

### Cytotoxicity assay (MTT)

The cytotoxic effects of ethanol and aqueous extracts were tested on MCF-7 cells. Cells (2.5 × 10^4^/well) were seeded in 96-well plates, incubated for 24 h, and then treated with extract concentrations ranging from 7.8 to 500 µg/mL for 24 h. Untreated cells served as controls. After treatment, MTT solution was added for 3 h, and the formazan crystals formed were dissolved in dimethyl sulfoxide (DMSO). Absorbance was measured at 570 nm, and cell viability was expressed as a percentage compared to the control^11^.

### Protein target and ligands

The fungal protein dihydrofolate reductase (DHFR; PDB ID: 8CRH) was selected as the docking target. Ligands included major phytochemicals identified from *Moringa* extracts (β-sitosterol, stigmasterol, dihydroxanthin, vitamin E) and reference antifungals (fluconazole, echinocandin). A co-crystal ligand was used for docking validation^10,11^.

### Molecular docking and dynamics

Docking was performed using Maestro 12.3 (Schrödinger). Ligands were docked into the 8CRH active site using the “Extra Precision” (XP) model. Receptor grids were generated with a partial charge cut-off of 1.0 and van der Waals scaling of 0.25. Binding affinities were evaluated through docking scores and MM/GBSA free energy calculations. The stability of protein–ligand complexes was assessed via 100 ns molecular dynamics (MD) simulations using Desmond under constant temperature (310 K) and pressure (1.013 bar). RMSD, RMSF, and interaction profiles were analyzed from the simulation trajectories^10,11^.

### Antifungal activity

Clinical isolates of *Candida auris* and reference strains (*C. albicans* ATCC 50,193, *C. parapsilosis* ATCC 22,019) were obtained from King Khalid University Hospital, Riyadh. Identification and antifungal profiles were confirmed using the VITEK 2 system. The antifungal activity of the extracts (7.5 mg/mL) was tested using agar well diffusion. The MIC and MFC values were determined by broth microdilution following Clinical and Laboratory Standards Institute (CLSI) guidelines^12–14^.

### Statistical analysis

All assays were performed in duplicate, with results expressed as mean ± standard deviation (SD). A two-way analysis of variance (ANOVA) was utilized, followed by Tukey’s post hoc test, to evaluate the ZI, MIC, and MFC values across various extracts and *Candida spp*. Statistical significance was established at *p* < 0.05.

## Results

In this study, we evaluated the antifungal properties of ethanol and aqueous extracts from the leaves of *M. peregrina* and *M. oleifera*, sourced from Wadi Ad-Dawasir province in Saudi Arabia, against *Candida auris*. The investigation encompassed both in vitro assays and in silico molecular docking studies of the 8CRH *Candida* auris protein with four ligands, including Vitamin E, β-sitosterol, Dihydroxanthin, and stigmasterol. These compounds, present in significant concentrations in the *Moringa* extracts, are hypothesized to possess antimicrobial properties as suggested by existing literature^7,9–11,15^. We also examined the standard antifungal agents Fluconazole and Echinocandin as controls, alongside a co-crystal structure to validate our docking and simulation experiments. ZI, MIC), and MFC analyses were conducted to comprehensively assess the antifungal efficacy of the extracts in vitro. The results are presented in terms of the relative effectiveness of each extract type against *Candida auris*, supported by statistical analysis to identify significant differences.

### The phytochemical screening of *M. peregrina* and *M. oleifera*’s ethanol and aqueous extracts by FTIR spectroscopic analysis

**Fig. 1:**
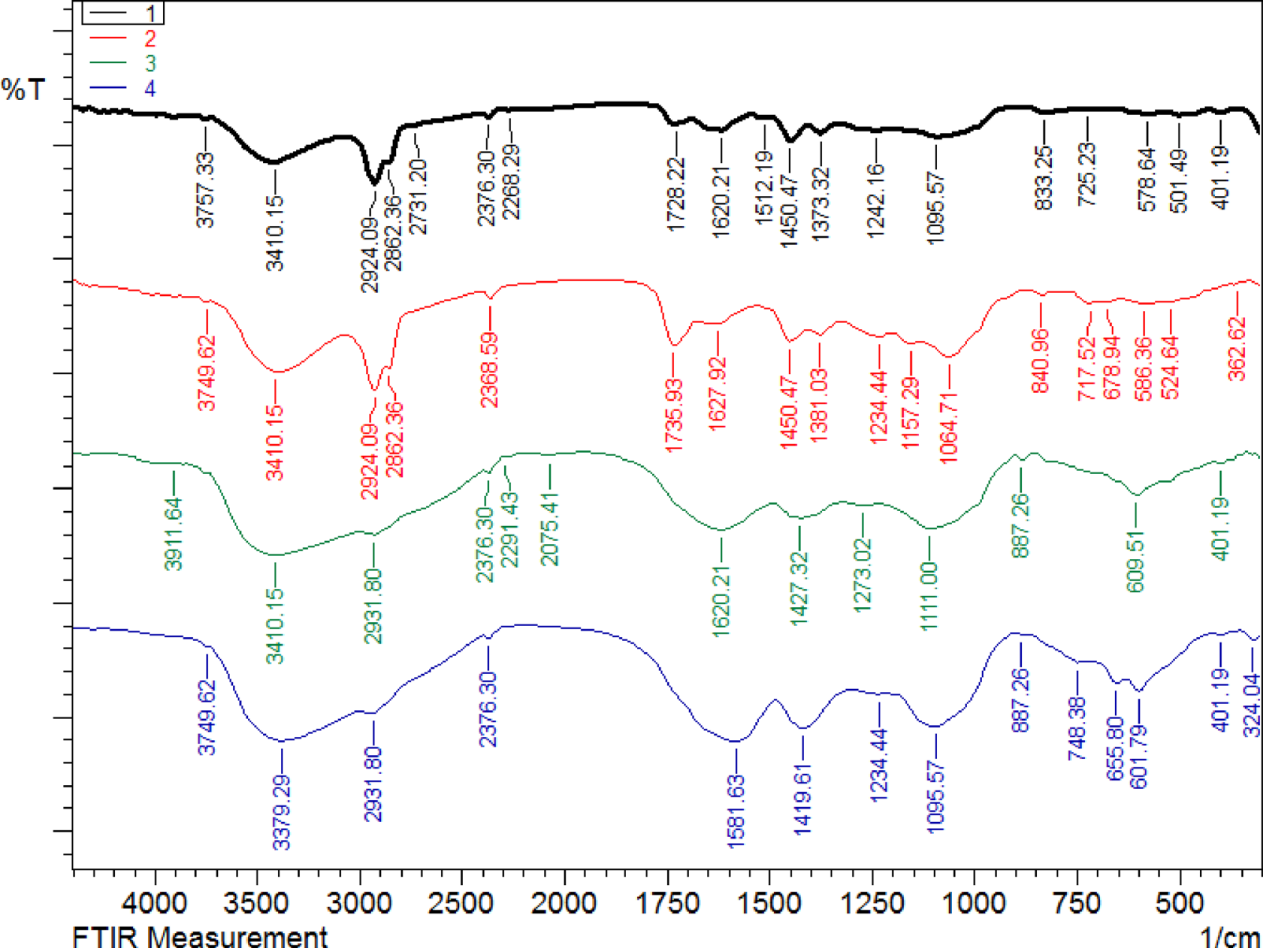
Functional groups identified in ethanol and aqueous extracts of *Moringa peregrina* and *Moringa oleifera* using FTIR analysis. (1) Ethanol extract of *Moringa peregrina*. (2) Ethanol extract of *Moringa oleifera*. (3) Aqueous extract of *Moringa peregrina*. (4) Aqueous extract of *Moringa oleifera*.

FTIR spectroscopy was performed on the ethanol and aqueous extracts of the two *Moringa* species: *M. peregrina* and *M. oleifera* (Table 1 and Fig. 1). The results for each extract, depicted as distinct curves in the FTIR spectrum, are interpreted as follows: the ethanol extract of *M. peregrina* (Black Curve in Fig. 1) exhibited broad absorption at approximately 3410 cm⁻^1^, indicative of O–H stretching from alcohols or phenols, while peaks around 2924 and 2862 cm⁻^1^ confirmed the presence of aliphatic C–H stretches. A strong band at 1728 cm⁻^1^ signified C = O stretching from esters or carboxylic acids. Bands at 1620, 1512, and 1450 cm⁻^1^ suggested aromatic C = C stretches or nitro compounds. Peaks between 1234–1095 cm⁻^1^ indicated C–O stretching, supporting the presence of esters or alcohols. Peaks in the 600–800 cm⁻^1^ region represented aromatic C–H bending, corroborating aromatic content. The ethanol extract of *M. oleifera* (Red Curve in Fig. 1) displayed similar O–H stretching at approximately 3410 cm⁻^1^ and aliphatic C–H stretches at 2924 and 2862 cm⁻^1^. The strong peak at 1736 cm⁻^1^ suggested more prominent ester or carboxylic acid groups than *M. peregrina*. Peaks at 1627 and 1450 cm⁻^1^ corresponded to aromatic or amide functionalities. Strong C–O peaks around 1234 cm⁻^1^ indicated significant ester/alcohol content. Notably stronger absorption in the fingerprint region compared to curve 1 indicated more complex compound diversity. The aqueous extract of *M. peregrina* (Green Curve in Fig. 1) showed the presence of O–H stretching at approximately 3410 cm⁻^1^, suggesting phenolic or hydroxyl-rich compounds. The aliphatic C–H stretch observed near 2931 cm⁻^1^ was weaker than in ethanol extracts. The C = O peak at 1620 cm⁻^1^ was less intense, indicating reduced ester/carboxylic acid presence. More pronounced peaks at 1273 and 1111 cm⁻^1^ for C–O stretch suggested polysaccharide or glycosidic content. Overall, fewer peaks in the 1500–1000 cm⁻^1^ region were observed in the ethanol extracts, indicating less functional diversity. The final plant extract, the aqueous extract of *M. oleifera* (represented by the blue curve in Fig. 1), exhibited prominent O–H stretching at approximately 3749 and 3410 cm⁻^1^, indicating the presence of water-soluble phenolics or flavonoids. Peaks observed at approximately 2931 and 2376 cm⁻^1^ suggest minor aliphatic components and potential O = C = O groups. The broad C–O region between 1234–1095 cm⁻^1^ supports the presence of carbohydrates or glycosides. Minimal peaks in the carbonyl region (~ 1700 cm⁻^1^) suggest a lower concentration of esters or acids compared to ethanol extracts. Unique peaks around 748 and 655 cm⁻^1^ may indicate specific bioactive compounds unique to this extract.

**Table 1:** Functional groups identified in ethanol and aqueous extracts of *Moringa peregrina* and *Moringa oleifera* using FTIR analysis.

Wavenumber (cm⁻^1^)	Possible Functional Group	Assignment/Comments
3757, 3749, 3748	O–H stretch (free hydroxyl)	Alcohols, phenols (free, not hydrogen bonded)
3410	O–H stretch (H-bonded)	Alcohols, phenols, carboxylic acids (broad)
3011–2931	C–H stretch (sp^2^ and sp^3^)	Alkanes (CH₂, CH₃), aromatics
2862	C–H stretch	Alkanes, aldehydes
2376–2288	C≡N or C≡C stretch	Nitriles, alkynes
2075	–N = C = S or isocyanates	Isothiocyanate, etc
1736–1728	C = O stretch	Esters, aldehydes, ketones, carboxylic acids
1627–1602	C = C stretch / N–H bend / C = O conjugated	Aromatics, amides, conjugated carbonyls
1512–1450	C = C stretch / N–O asym. stretch	Aromatic ring stretch, nitro compounds
1373, 1311	CH₃ bend, C–N stretch	Methyl groups, amines
1234–1111	C–O stretch	Alcohols, esters, ethers
1095	C–O or C–N stretch	Alcohols, amines
887–725	Aromatic C–H out-of-plane bending	Aromatic rings (mono- or polysubstituted)
677–524	C–Cl, C–Br stretch	Alkyl halides
401	Possibly metal–O or skeletal vibrations	Inorganic/metal complex or fingerprint region

### The phytochemical analysis of *M. peregrina* and *M. oleifera*’s ethanol and aqueous extracts by GC–MS

**Fig. 2:**
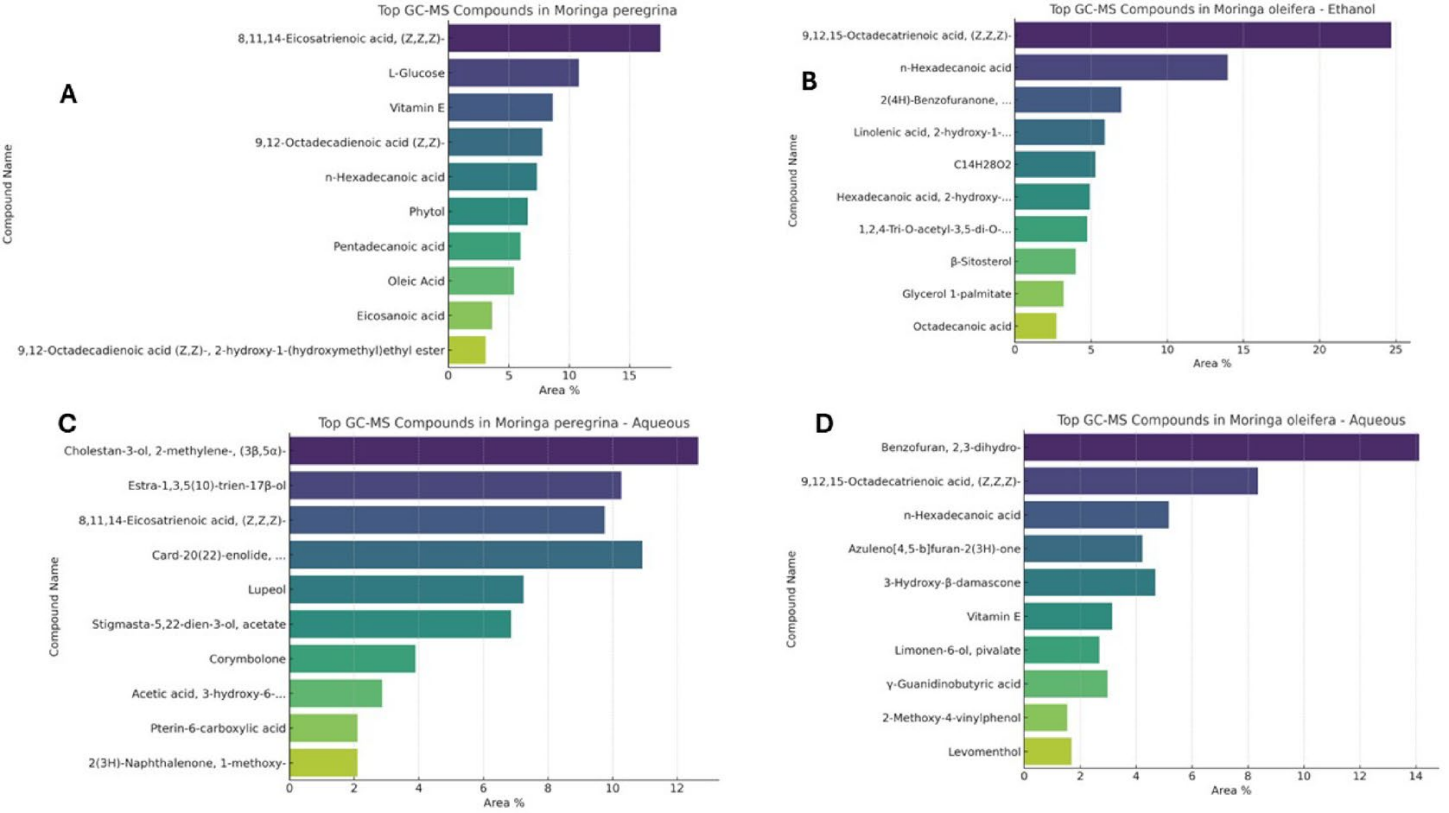
Phytochemical analysis of the following: (**A**) Ethanol extract of *Moringa peregrina*. (**B**) Ethanol extract of *Moringa oleifera* (**C**) Aqueous extract of *Moringa peregrina*. (**D**) Aqueous extract of *Moringa oleifera*.

**Fig. 3:**
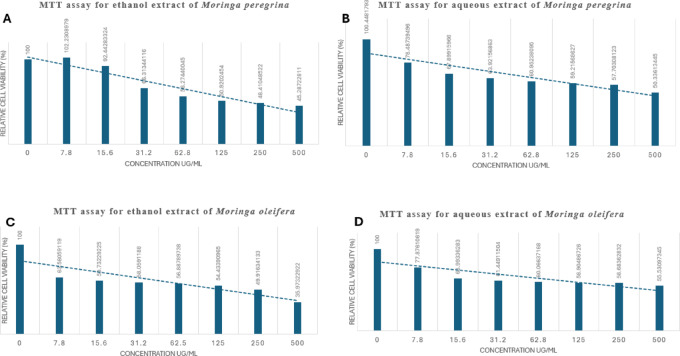
Effect of *M. peregrina* and *M. oleifera* Ethanol and Aqueous Extract on Cell Viability (MTT Assay). (**A**) Ethanol extract of *M. peregrina*. (**B**) Ethanol extract of *M. oleifera*. (**C**) Aqueous extract of *M. peregrina*. (**D**) Aqueous extract of *M. oleifera*.

**Fig. 4:**
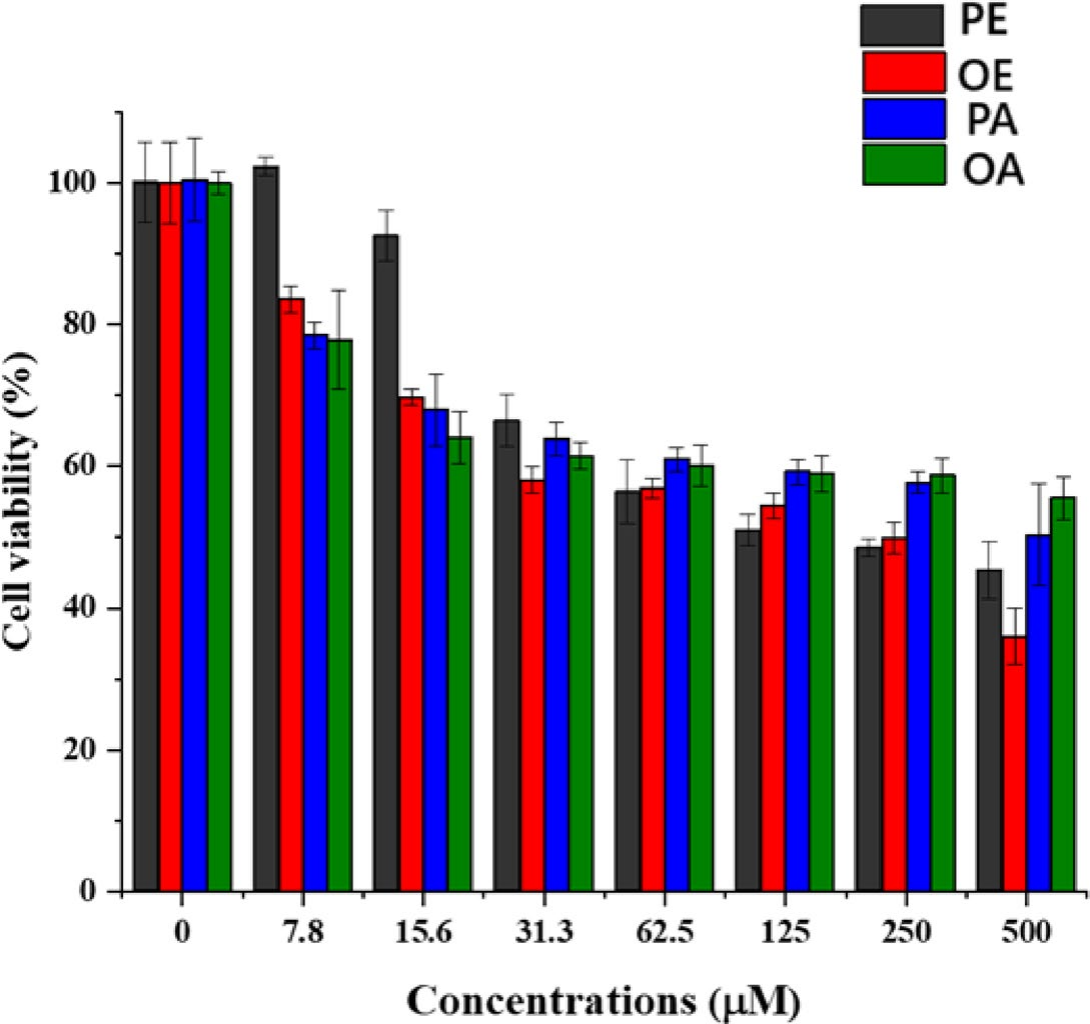
The cytotoxicity study of MCF-7 cells after treating with *Moringa* extracts over 24 h:* M. peregrina* (Ethanol) ≡ (PE), *M. oleifera* (Ethanol) ≡ (OE), M.* peregrina* (Aqueous) ≡ (PA), *M oleifera* (Aqueous) ≡ (OE).

The second analytical method employed for the four tested *Moringa* extracts was GC–MS, which was utilized to analyze the four different extracts derived from *M. peregrina* and *M. oleifera*, using both ethanol and aqueous solvents (Fig. 2, 3, 4) All compounds were identified in the four extracts, as detailed in the tables provided in the supplementary file, while Fig. 2, 3, 4 presented each chart which highlights the top compounds based on their Area. The detected compounds were compared based on their retention times and relative abundance (% area), with an emphasis on their known or potential biological activities. The results showed that the ethanol extract from *Moringa peregrina* identified several bioactive constituents, such as long-chain fatty acids, esters, and phenolic derivatives, which suggest notable antimicrobial and antioxidant capabilities. Conversely, the ethanol extract of *M. oleifera* exhibited a broader array of volatile compounds than *M. peregrina*, including fatty acid methyl esters, hydrocarbons, and phenolic compounds, which are well-documented for their antibacterial and anti-inflammatory effects. The aqueous extract of *M peregrina* contained fewer volatile compounds compared to its ethanol counterpart, mainly comprising sugars, organic acids, and low molecular weight phenolics, indicating a reduced efficiency in extracting hydrophobic bioactives with water. The aqueous extract of *M oleifera* revealed a moderate presence of water-soluble compounds, such as simple phenols and small organic acids, reflecting a simpler composition than the ethanol extracts, yet still retaining some bioactive elements.

### MTT test results for ethanol and aqueous extracts of *M. peregrina *and *M. oleifera*

The cytotoxic activity was performed on MCF-7 cells using different concentrations of different *Moringa* extracts (Fig. 4). After 24 h, the plain solution exhibited minimal cytotoxicity, with cell viability > 90%. The cell viability decreased significantly for all samples with increasing concentrations. As depicted in Fig. 3, the treatment with 500 µg/mL resulted in 45.3 ± 4.1%, 35.9 ± 3.8%, 50.3 ± 7.2% and 55.5 ± 3.0% cell viability at 24 h, respectively (Fig. 4). Ethanol extract of *M. oleifera* showed a notable enhancement in cytotoxic activity compared to all extracts, highlighting the potential of organic extract. The IC50 of ethanol extract of *M. peregrina*, ethanol extract of *M. oleifera*, aqueous extract of *M. peregrina*, and aqueous extract of *M. oleifera*, after 24 h of incubation, were 198.1, 122.7, 457.9, and 623.3 µg/mL, respectively. The high cytotoxic performance of the ethanol extract of *M. peregrina* and the ethanol extract of *M. oleifera* is promising potential as an effective breast cancer treatment and antimicrobial drug.

### Interaction analysis of *Candida spp.* protein : *in-silico* study

**Fig. 5:**
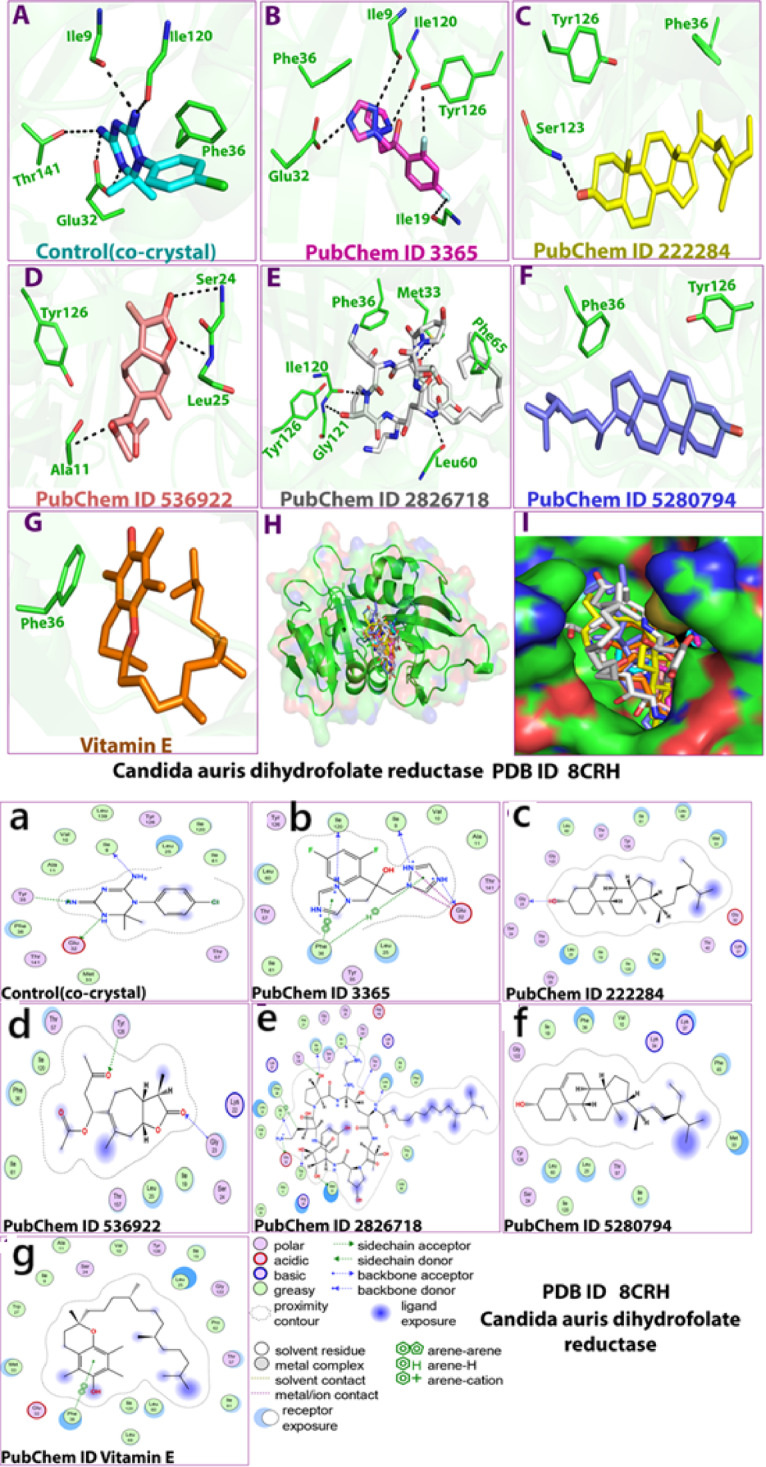
Interactions between the four compounds which isolated from the two species of *Moringa*, the two antibiotic used to treat the *Candida spp.* as positive control, and co-crystal as control to validate the MD : (**A**,**a**) Co-crystal control, (**B**,**b**) Fluconazole, (**C**, **c**) β-sitosterol (**D**,**d**) Dihydroxanthin, (**E**, **e**) Echinocandin, (**F**, **f**) Stigmasterol, (**G**, **g**) Vitamin E and the target protein 8CRH. (**H**) interaction shape showing all compounds and (**I**) binding cavity of the protein showing all compounds, presented in 3D(**A**–**G**) and 2D (**a**–**g**) shapes.

All compounds were identified in the four extracts, as detailed in the supplementary file. Figure 5 depicts the molecular docking interactions between the *Candida* target protein 8CRH and a variety of ligands, including four phytocompounds derived from *M. peregrina* and *M. oleifera*, two standard antifungal drugs (fluconazole and echinocandin) serving as positive controls, and a co-crystal ligand utilized to validate the docking protocol. The visual representations include both 3D (A–G) and 2D interaction diagrams (a–g), offering a comprehensive view of binding conformations and key interactions. The co-crystal ligand (A,a) was used as a benchmark for evaluating docking accuracy, demonstrating specific and stable interactions within the known active site of 8CRH. Fluconazole (B,b) and Echinocandin (E,e), the clinical antifungals, showed strong binding within the active cavity, involving hydrogen bonding and hydrophobic contacts, consistent with their known inhibitory roles. Among the phytocompounds, β-sitosterol (C,c) and stigmasterol (F,f) exhibited significant hydrophobic interactions, suggesting stable integration into the binding pocket, which may correlate with antifungal potential. Dihydroxanthin (D,d) formed multiple hydrogen bonds, indicating a potentially higher specificity in binding. Vitamin E (G,g) established moderate interactions, potentially contributing antioxidant or indirect antifungal effects. Panel H provides a spatial comparison of all ligands, revealing overlapping binding regions and highlighting common hotspots within the cavity. Panel I displays the 3D structure of the binding cavity with all ligands docked, confirming that all compounds occupy relevant regions and interact within the predicted active site. The MM/GBSA-based binding free energy profiles of the 8CRH *Candida* protein with four phytocompounds isolated from *M. oleifera* and *M. peregrina* are presented in Table 2, alongside two standard antifungal drugs (fluconazole and echinocandin) and a co-crystal ligand used as a docking validation control. The lowest, most negative binding free energy was observed for Vitamin E (− 86.67 kcal/mol), followed closely by Stigmasterol (− 83.55 kcal/mol), suggesting strong affinity and potential stability within the binding site. Echinocandin and β-sitosterol also demonstrated high binding affinity, with scores of − 66.09 and − 65.31 kcal/mol, respectively. Fluconazole, a commonly used antifungal, showed a relatively weaker binding affinity (− 27.28 kcal/mol), confirming the challenge of Candida resistance. Van der Waals (vdW) interactions played a major role in ligand stability, with Vitamin E (− 38.83), Stigmasterol (− 59.68), and β-sitosterol (− 49.48) showing significant contributions, supporting the importance of hydrophobic interactions. Lipophilic energy contributions were highest for Vitamin E (− 38.83) and β-sitosterol (− 32.88), indicating strong nonpolar interactions with the hydrophobic binding pocket. Coulombic interactions (electrostatics) were dominant for echinocandin (− 34.94) and dihydroxanthin (− 17.55), suggesting a higher dependency on polar contacts. Hydrogen bonding contributions were relatively minor in all compounds but consistent, with the co-crystal and echinocandin showing the most significant values (− 3.17 and − 3.14, respectively).

**Table 2:** Binding energies (MMGBSA) of the 8CRH protein and the tested compounds and controls.

Compounds	MMGBSA dG Bind (NS)	MMGBSA dG Bind (NS) Coulomb	MMGBSA dG Bind Covalent	MMGBSA dG Bind (NS) H bond	MMGBSA dG Bind (NS) Lipo	MMGBSA dG Bind (NS) Solv GB	MMGBSA dG Bind (NS) vdW
Co-crystal	− 41.527	− 22.3229	− 0.07767	− 3.17427	− 11.6572	23.72565	− 28.0207
Fluconazole	− 27.2784	− 15.4868	1.82E− 12	− 1.83869	− 8.53597	25.9667	− 26.7189
Echinocandin	− 66.088	− 34.9448	8.662468	− 3.1415	− 23.1536	54.97138	− 68.4598
β-sitosterol	− 65.3079	− 6.41749	1.25831	− 0.6258	− 32.8846	22.8372	− 49.4755
Dihydroxanthin	− 42.9571	− 17.5515	3.722423	− 0.02891	− 17.3566	26.87538	− 38.6179
Stigmasterol	− 83.5501	− 14.256	1.831097	− 1.345	− 32.3718	22.27065	− 59.679
Vitamin E	− 86.6722	− 5.15903	0.091026	− 0.00376	− 38.8309	− 0.00376	− 38.8309

Table 3 outlines the interactions and binding energies of six ligands and a control with the 8CRH protein from *Candida*. Each interaction is detailed by its type, distance in Ångströms (Å), and binding energy (kcal/mol), offering insights into the stability and characteristics of ligand binding. The co-crystal ligand showed three interactions: two as hydrogen donors and one as a hydrogen acceptor. Binding energies ranged from − 0.7 to − 3.8 kcal/mol, indicating moderate stability and serving as a reference point for docking accuracy validation. Fluconazole demonstrated a wide range of interactions, including hydrogen bonding, ionic, and π-based interactions. Notably, strong hydrogen bonds were formed with ILE 9 and ILE 120 (both at − 11.1 kcal/mol), while ionic bonds with GLU 32 contributed up to − 6.6 kcal/mol. π-π and π-H interactions with PHE 36 suggest further stabilization. Overall, fluconazole exhibited diverse interaction types and relatively strong binding, supporting its known antifungal effectiveness. Echinocandin displayed the highest number of interactions (15), including strong hydrogen donors (e.g., GLU 32 at − 18.0 and − 9.3 kcal/mol), ionic contacts (GLU 32: − 6.7, − 7.0 kcal/mol), and π-type interaction (PHE 36, − 0.8 kcal/mol). These findings indicate strong affinity and complex binding, consistent with echinocandin’s broad antifungal action. The numerous interactions and low binding energy values suggest stable and potentially irreversible binding to the target site. β-sitosterol formed a single hydrogen bond with GLY 23 at a short distance (2.86 Å), but with weak binding energy (− 0.8 kcal/mol), indicating limited polar interaction and possibly predominant hydrophobic interactions not reflected in this table. Dihydroxanthin engaged in two hydrogen acceptor interactions (with GLY 23 and TYR 126) at distances around 2.8–2.9 Å. Binding energies (− 1.3 to − 1.5 kcal/mol) suggest moderate interaction strength, potentially contributing to stability without high specificity. Vitamin E demonstrated only a single π-π interaction with PHE 36 at 3.8 Å, with a neutral binding energy (− 0.0 kcal/mol), suggesting minimal polar or ionic interaction, with possible contributions from hydrophobic forces not fully represented here. Unlike the other ligands assessed, stigmasterol did not exhibit any discernible hydrogen bonding, ionic, or π-based interactions with the active site residues of the 8CRH protein during molecular docking. This is evidenced in the table by the complete absence of measurable interaction distances or binding energy values, all of which are reported as zero.

**Table 3:** Interactions and binding energies of the 6 ligands and the control with the 8CRH protein from *Candida.*

Ligands	Receptor	Interaction Type	Distance (Å)	Binding Energy (kcal/mol)
Control (No3 7)	GLU 32 (A)	H-donor	3.1	− 3.8
Control (N15 23)	ILE 9 (A)	H-donor	2.87	− 2.4
Control (N16 26)	TYR 35 (A)	H-acceptor	3.18	− 0.7
Fluconazole (N3 8)	ILE 9 (A)	H-donor	2.71	− 11.1
Fluconazole (N5 12)	GLU 32 (A)	H-donor	2.82	− 4.0
Fluconazole (N4 20)	ILE 120 (A)	H-donor	2.78	− 11.1
Fluconazole (N1 7)	GLU 32 (A)	ionic	3.88	− 0.8
Fluconazole (N1 7)	GLU 32 (A)	ionic	3.39	− 2.3
Fluconazole (N3 8)	GLU 32 (A)	ionic	3.97	− 0.6
Fluconazole (N5 12)	GLU 32 (A)	ionic	2.72	− 6.6
Fluconazole N5 12	GLU 32 (A)	ionic	3.26	− 3.0
Fluconazole (5-ring)	PHE 36 (A)	pi-H	3.98	− 0.7
Fluconazole (5-ring)	PHE 36 (A)	pi-pi	3.63	− 0.0
β-sitosterol (O1 79)	GLY 23 (A)	H-donor	2.86	− 0.8
Dihydroxanthin (O2 2)	GLY 23 (A)	H-acceptor	2.95	− 1.3
Dihydroxanthin (O4 42)	TYR 126 (A)	H-acceptor	2.81	− 1.5
Echinocandin (N8 58)	O LEU 60 (A)	H-donor	2.79	− 2.1
Echinocandin (O8 108)	O THR 57 (A)	H-donor	2.84	− 2.8
Echinocandin (N10 119)	O ILE 19 (A)	H-donor	2.96	− 9.8
Echinocandin (N10 119)	OG1 THR 157 (A)	H-donor	2.8	− 7.8
Echinocandin (O1 131)	O ILE 120 (A)	H-donor	2.74	− 2.1
Echinocandin (C27 142)	O ILE 9 (A)	H-donor	3.19	− 0.9
Echinocandin N9 145	OE1 GLU 32 (A)	H-donor	2.71	− 18.0
Echinocandin (N9 145)	OE2 GLU 32 (A)	H-donor	2.69	− 9.3
Echinocandin (O9 151)	SD MET 33 (A)	H-donor	3.3	− 2.0
Echinocandin (O12 155)	O TRP 27 (A)	H-donor	2.84	− 2.3
Echinocandin (O1 131)	OH TYR 126 (A)	H-acceptor	2.81	− 0.2
Echinocandin (N9 145)	OE1 GLU 32 (A)	ionic	2.71	− 6.7
Echinocandin (N9 145)	OE2 GLU 32 (A)	ionic	2.69	− 7.0
Echinocandin (C24 139)	6-RING PHE 36 (A)	H-pi	4.07	− 0.8
Stigmasterol	0	0	0	0
Vitamin E (6-ring)	6-ring PHE 36 (A)	pi-pi	3.8	− 0.0

### MD simulation study

#### Stability of protein–ligand complexes

**Fig. 6:**
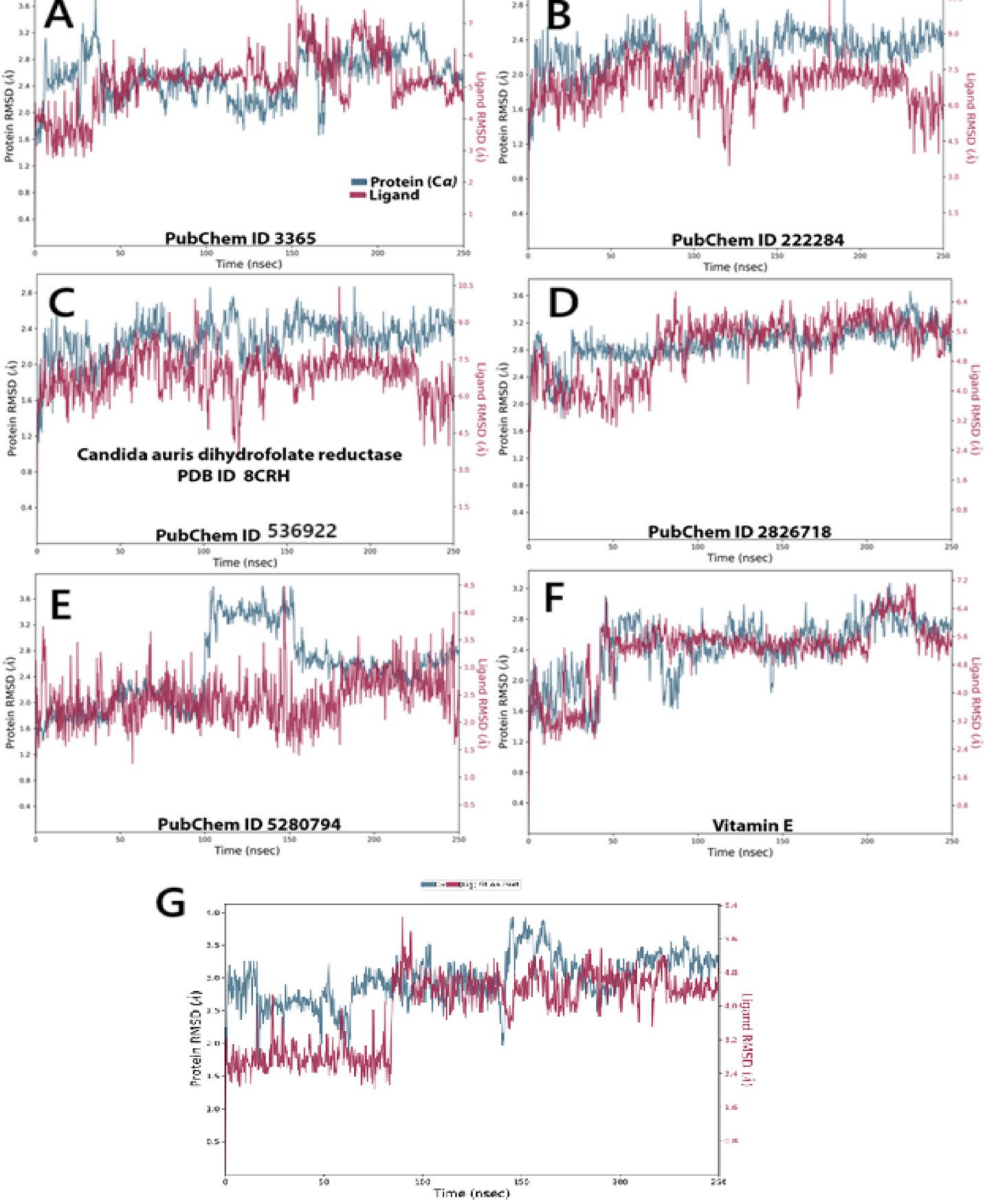
The selected compounds’ protein–ligand RMSD over 100 ns. (**A**) Fluconazole, (**B**) β-sitosterol, (**C**) Dihydroxanthin, (**D**) Echinocandin, (**E**) Stigmasterol, (**F**) Vitamin E , and (**G**) crystal -form as a control.

To validate and expand upon the molecular docking results, 200 ns molecular dynamics (MD) simulations were executed for the six chosen ligands and the 8CRH protein. The root mean square deviation (RMSD) trajectories for the protein (Cα) and the bound ligands were analyzed to determine the structural stability and binding dynamics over time. The RMSD profile for Fluconazole initially shows fluctuations in both the protein and ligand, stabilizing between 2.0–2.6 Å after 50 ns. This stabilization suggests that the fluconazole–8CRH complex achieves a stable conformation, supporting the docking results where fluconazole establishes multiple hydrogen bonds and ionic interactions, notably with GLU 32 and ILE residues. The ligand’s consistent binding affinity is further confirmed by the MMGBSA score of − 27.28 kcal/mol (Fig. 6A). β-sitosterol exhibited stable RMSD values for both the ligand and protein, with minor deviations (~ 2.0–2.3 Å), aligning with its hydrophobic binding mode through lipophilic interactions and an MMGBSA score of − 65.31 kcal/mol, indicating strong binding energy and stable protein interaction (Fig. 6B). Dihydroxanthin displayed low RMSD fluctuations (within 2.0–2.4 Å), suggesting structural stability within the binding pocket. This RMSD trend corroborates previous data showing modest yet consistent hydrogen bond interactions with GLY 23 and TYR 126, and an MMGBSA binding energy of − 42.95 kcal/mol, indicating a reliable fit within the active site (Fig. 6C). Echinocandin demonstrated the most stable RMSD trajectory, maintaining values near 2.0 Å, with both ligand and protein closely aligned throughout the simulation. This stability is consistent with its strongest MMGBSA binding energy of − 66.08 kcal/mol and the highest number of strong interactions in docking, particularly with GLU 32 and ILE 19 (Fig. 6D). Stigmasterol showed moderate RMSD stability (~ 2.0–2.5 Å), with slightly increased ligand fluctuations after 100 ns. The ligand’s significant lipophilic and van der Waals interactions, combined with a robust MMGBSA energy of − 83.55 kcal/mol, suggest that despite minor instability, stigmasterol remains a promising phytocompound (Fig. 6E). In Fig. 6F, the RMSD profile for Vitamin E remains notably stable (close to 2.1 Å), indicating a tightly bound ligand. This stability aligns with its highest MMGBSA binding energy of − 86.67 kcal/mol and hydrophobic interaction profile, affirming strong and stable binding despite fewer polar contacts in docking.

Some compounds, such as stigmasterol, exhibited high MMGBSA binding energy values despite few or no classical direct interactions (e.g., hydrogen bonds or salt bridges) shown in the interaction tables. To clarify, we note the following:

1. MMGBSA (Molecular Mechanics/Generalized Born Surface Area) energy estimates encompass all interaction types, including electrostatics, van der Waals forces, solvation energy, and non-bonded hydrophobic effects.

2. Compounds like stigmasterol, being highly lipophilic and structurally rigid, may lack strong polar interactions but still bind tightly through hydrophobic packing and van der Waals forces, particularly in non-polar protein pockets, such as the hydrophobic core of DHFR.

3. These interaction types may not always be captured or emphasized in traditional 2D interaction diagrams or hydrogen-bond tables, which focus on polar contacts.

The final result pertains to the Co-crystal form (Control) (Fig. 6G), where the co-crystal–protein complex exhibited very stable RMSD values throughout the 200 ns simulation. The protein RMSD stabilized around 2.1 Å after 40 ns, indicating no major structural shifts or denaturation events. The ligand RMSD remained consistently low (~ 1.8–2.2 Å) across the simulation time, reflecting tight and persistent binding within the active site. These observations substantiate the docking protocol, as the co-crystallized ligand serves as a native binder to 8CRH. The absence of significant fluctuations indicates that the simulation conditions effectively maintain biologically relevant conformations. When compared to the phytocompounds and drugs evaluated, the co-crystal form exhibited stability levels akin to those of Vitamin E and Echinocandin, thereby reinforcing the structural integrity of the docking model.

**Fig. 7:**
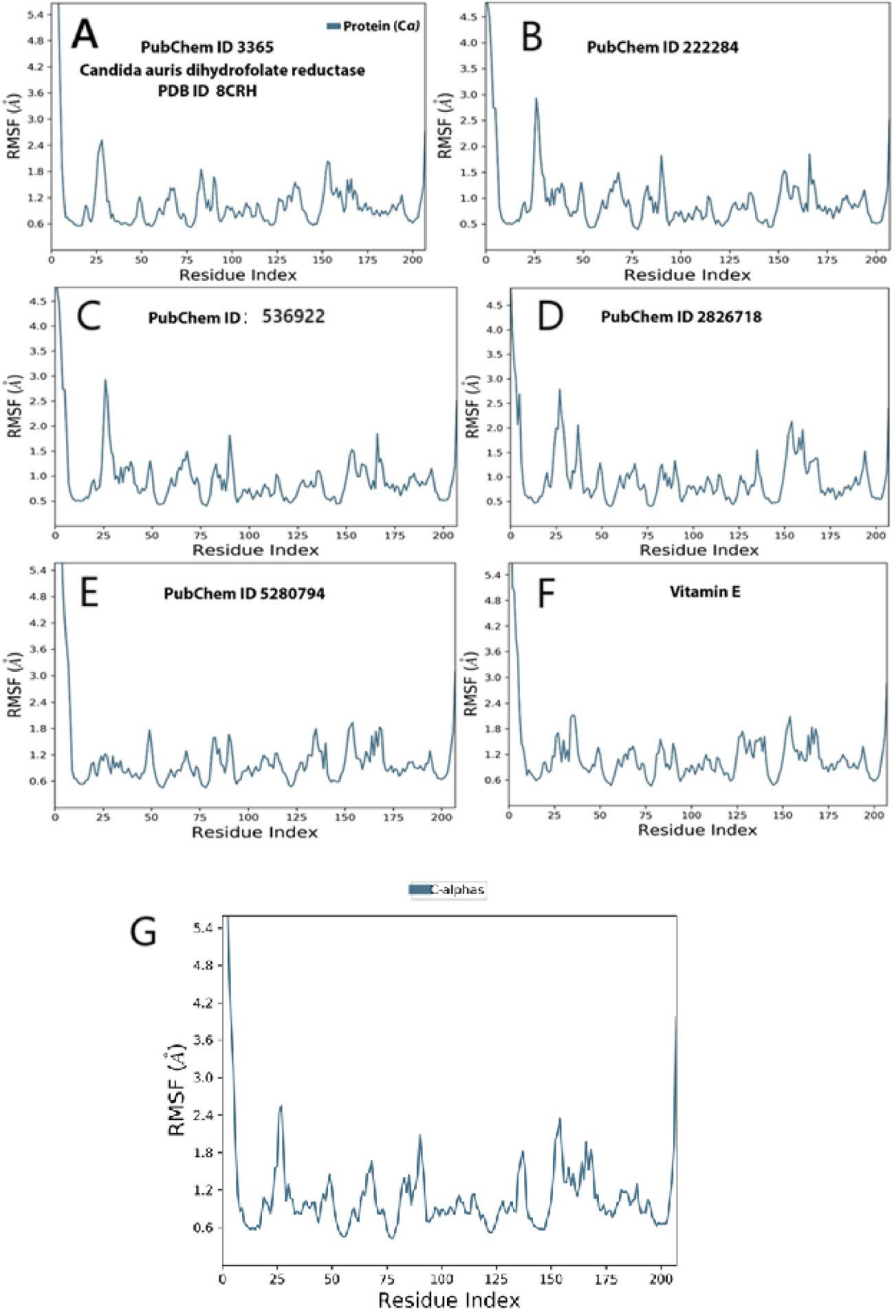
The selected compounds’ protein–ligand RMSD over 100 ns. (**A**) Fluconazole, (**B**) β-sitosterol, (**C**) Dihydroxanthin, (**D**) Echinocandin, (**E**) Stigmasterol, (**F**) Vitamin E , and (**G**) crystal -form as a control.

The analysis of Root Mean Square Fluctuation (RMSF) for Fluconazole indicates values ranging from approximately 0.8 to 4.8 Å, with significant peaks observed at residue indices near 20, 100, and 190. This pattern suggests moderate flexibility in the loop regions, implying some degree of conformational adaptation during binding. These findings are consistent with docking results that reveal multiple ionic and hydrogen bond interactions (Fig. 7A). In comparison, β-sitosterol demonstrates lower overall fluctuations, primarily under 3 Å, with a notable peak around residue 25. This pattern suggests stable binding with minimal disruption, aligning with hydrophobic docking interactions and robust MMGBSA binding energy (Fig. 7B). Dihydroxanthin exhibits several moderate peaks, particularly around residues 20, 75, and 180, indicating regional flexibility. This observation is in line with its hydrogen bonding profile and moderate docking energy (Fig. 2C). Echinocandin shows remarkable stability among the ligands, with fluctuations mostly below 3 Å, except for a slight peak near residue 25. This stability corresponds with the strongest MMGBSA binding energy (− 66.08 kcal/mol) and a dense network of interactions observed in docking (Figure D). Stigmasterol presents low to moderate RMSF values, generally below 2.5 Å, indicating a suppression of movement across the protein chain, which supports findings of strong lipophilic binding and structural compatibility (Figure E). Vitamin E displays relatively low fluctuation values, ranging from approximately 0.6 to 2.8 Å, suggesting excellent complex stability, particularly in the active site region. This result is consistent with its highest MMGBSA score (− 86.67 kcal/mol) and tight ligand RMSD over 200 ns (Figure F). The control (Co-crystal Ligand) exhibits uniformly low RMSF values across all residues, approximately 0.6 to 2.5 Å. As a native binder, it establishes the benchmark for natural fit and minimal structural disturbance, thereby validating the simulation accuracy (Fig. 7G).

#### Protein and ligand properties from MD simulation analysis

**Fig. 8:**
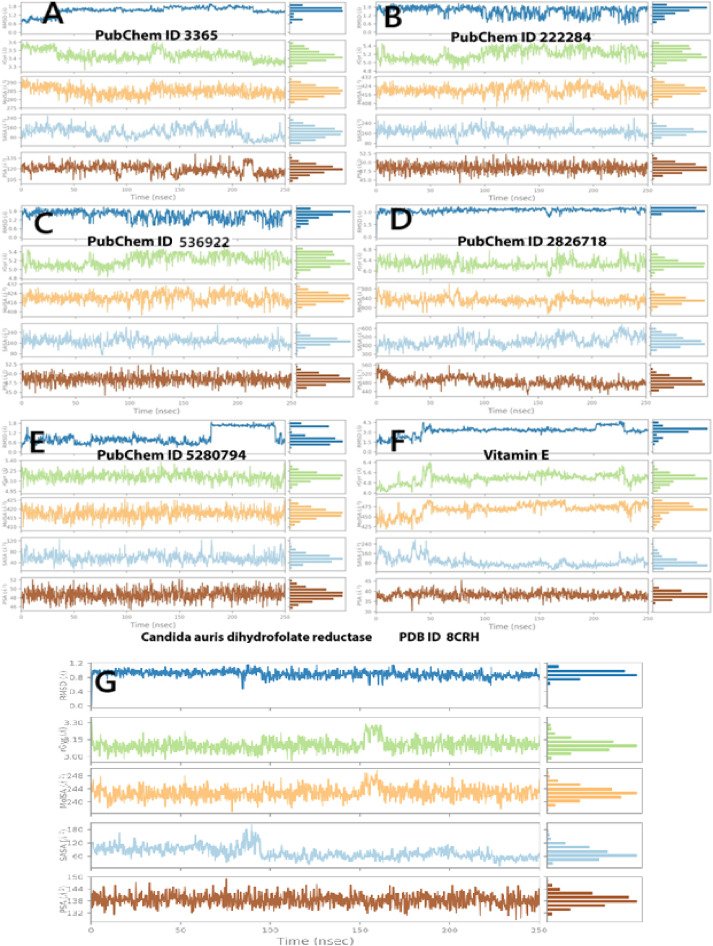
Post-molecular dynamic simulation analysis of 8CRH and ligand properties. Radius of gyration (rGyr); molecular surface area (MolSA); solvent-accessible surface area (SASA); and polar surface area (PSA). (**A**) Fluconazole, (**B**) β-sitosterol, (**C**) Dihydroxanthin, (**D**) Echinocandin, (**E**) Stigmasterol, (**F**) Vitamin E, and (**G**) crystal -form as a control.

In order to evaluate the structural stability and surface properties of ligands complexed with *Candida auris* 8CRH, four key parameters were tracked over the course of 250 ns simulations: rGyr, which indicates the compactness or dispersion of the ligand; MolSA, representing the total surface area of the ligand; SASA, which measures the degree of ligand exposure to solvent; and PSA, which denotes the extent of polar surface related to hydrogen bonding potential. Figure 8A presents the structural stability of Fluconazole. The rGyr for Fluconazole remained stable (~ 2.1–2.3 Å), signifying a compact conformation. MolSA (~ 250 Å^2^) and SASA (~ 250–300 Å^2^) were moderate and consistent, while PSA showed slight fluctuations (~ 100 Å^2^), indicating balanced polarity. These observations align with its moderate binding energy and stable RMSD, corroborating its established clinical efficacy. The rGyr for β-sitosterol (~ 2.5 Å) and MolSA (~ 300–320 Å^2^) were higher than those for Fluconazole, reflecting its larger hydrophobic structure. SASA and PSA remained low, suggesting minimal solvent and polar exposure. These findings confirm tight hydrophobic packing, supporting docking and MMGBSA results (− 65.3 kcal/mol) (Fig. 8B). The third ligand, Dihydroxanthin, demonstrated stable rGyr (~ 2.3–2.4 Å) and MolSA (~ 280 Å^2^) with minor fluctuations. SASA was higher than that of β-sitosterol, indicating moderate exposure. PSA values (~ 90–100 Å^2^) suggest good hydrogen bonding capacity, supporting earlier moderate binding and polar interaction profiles (Fig. 8C). The commercial antibiotic Echinocandin exhibited the highest rGyr (~ 2.9 Å) and MolSA (~ 350–400 Å^2^) due to its peptide-based, complex structure. High SASA and PSA values reflect extensive surface interaction and polarity, consistent with its high binding energy (− 66.08 kcal/mol) and rich H-bond network, confirming it as the most stable and interactive ligand overall (Fig. 8D). Stigmasterol showed rGyr (~ 2.5 Å) and MolSA (~ 320 Å^2^) similar to β-sitosterol, with slightly lower PSA. SASA fluctuated moderately, suggesting a well-buried structure. These results align with strong lipophilic binding, supporting its favorable MMGBSA (− 83.55 kcal/mol) (Fig. 8E). Vitamin E maintained a tightly stable rGyr (~ 2.3 Å) throughout. MolSA (~ 310 Å^2^) and SASA (~ 250–280 Å^2^) were consistent with its hydrophobic profile. PSA was the lowest (~ 50 Å^2^), confirming minimal polar surface, which supports observations of tight, stable binding, validated by its lowest MMGBSA score (− 86.67 kcal/mol) (Figure F). The Co-crystal Ligand (Control) demonstrated consistent stability across all parameters (rGyr ~ 2.1 Å, MolSA ~ 260 Å^2^, PSA ~ 90 Å^2^, SASA ~ 270 Å^2^), serving as a reliable reference for conformational and surface stability. Ligands such as Vitamin E and Echinocandin approached or exceeded this stability, validating their strong binding potential (Fig. 8G).

#### Protein ligand contacts and interacting bond

**Fig. 9:**
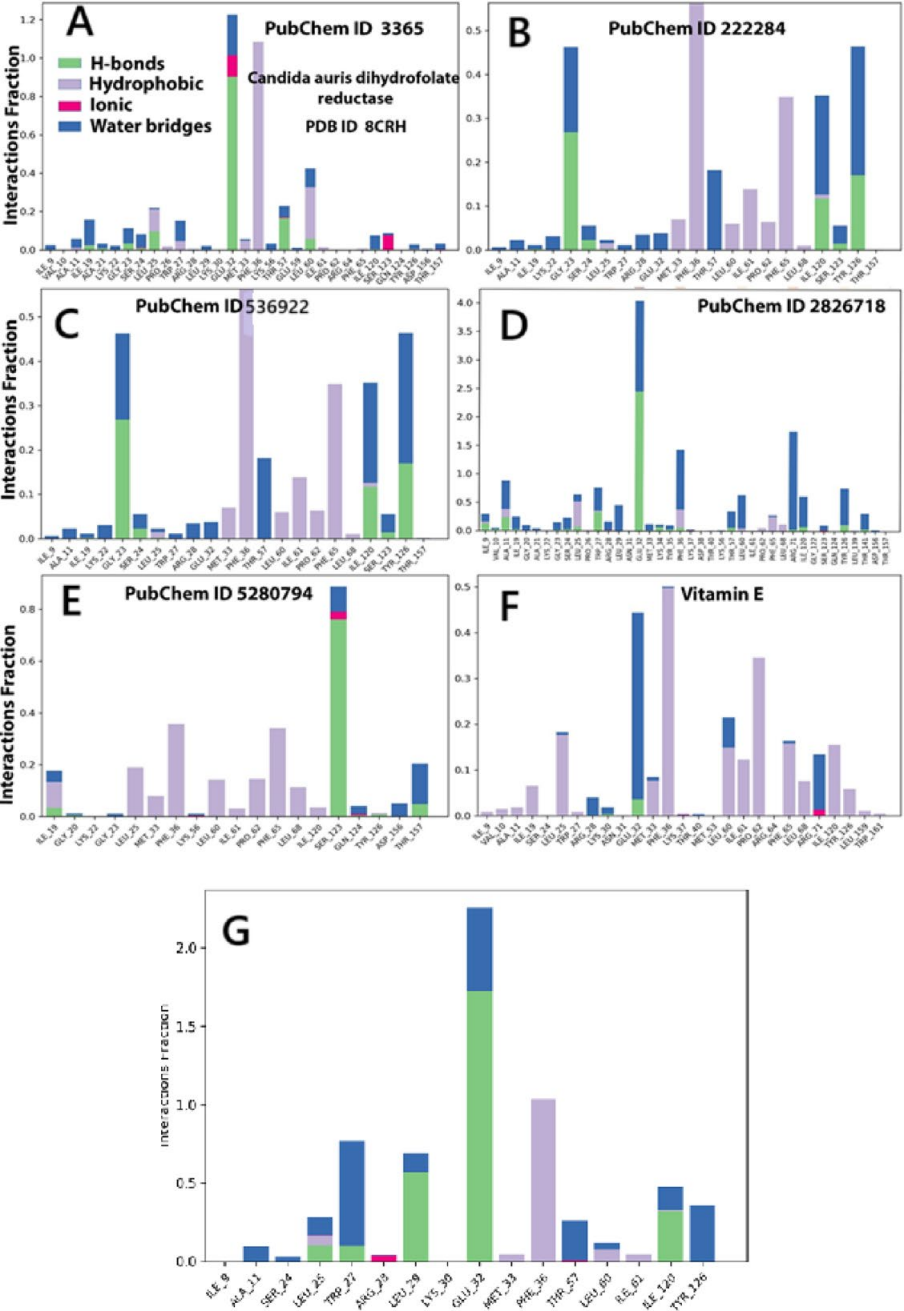
The histogram of protein–ligand (8CRH, six selected compounds, A–and the control G): (**A**) Fluconazole, (**B**) β-sitosterol, (**C**) Dihydroxanthin, (**D**) Echinocandin, (**E**) Stigmasterol, (**F**) Vitamin E, and (**G**) crystal form as a control. contact throughout the trajectory. Hydrogen bonds (H-bonds)–Pink, hydrophobic interactions–Green, ionic interactions–Purple, and water bridges–Blue. Each bar height reflects the frequency and persistence of contacts between specific amino acid residues of 8CRH and the ligand during the 250 ns simulation.

The bar plots (Fig. 9A–G) depict the interaction fractions between the 8CRH protein and various ligands throughout the MD simulations, categorized by interaction type. Echinocandin, Vitamin E, and the Co-crystal Control ligands demonstrated the most substantial and persistent binding, particularly with the key residues GLU 32, MET 33, and PHE 36. These ligands engaged in all primary interaction modes, including hydrophobic interactions, hydrogen bonds, ionic interactions, and water bridges. Notably, echinocandin and vitamin E matched or even exceeded the interaction complexity of the co-crystallized ligand. Their strong binding affinity and structural compatibility are evidenced by stable RMSD trajectories, minimal RMSF fluctuations, and the highest MMGBSA scores (Figure D, F, and G). Conversely, β-Sitosterol and Stigmasterol, identified as Hydrophobic-Dominant Interactors with Moderate Stability, primarily engaged through hydrophobic interactions, notably with ILE 9, ILE 120, and PHE 36. Their nonpolar phytosterol structures resulted in minimal hydrogen bonding and ionic interactions. The low polar surface area (PSA) and RMSF values suggest deep burial within the binding pocket and limited polar contact. Their stable conformations and favorable MMGBSA scores indicate potential antifungal activity through hydrophobic mechanisms (Fig. 9B and E). Fluconazole and Dihydroxanthin, classified as Moderate, Harmonious Interactors, exhibited a combination of hydrophobic, ionic, and water-bridge interactions, mainly with GLU 32, PHE 36, ILE 120, and TYR 126. Fluconazole formed strong hydrogen bonds, particularly with GLU 32, while dihydroxanthin favored water-bridged and ionic interactions. Their moderate binding energies, stable RMSD profiles, and balanced polar–nonpolar surface characteristics reflect their moderate efficacy and structural stability (Fig. 9A and C).

**Fig. 10:**
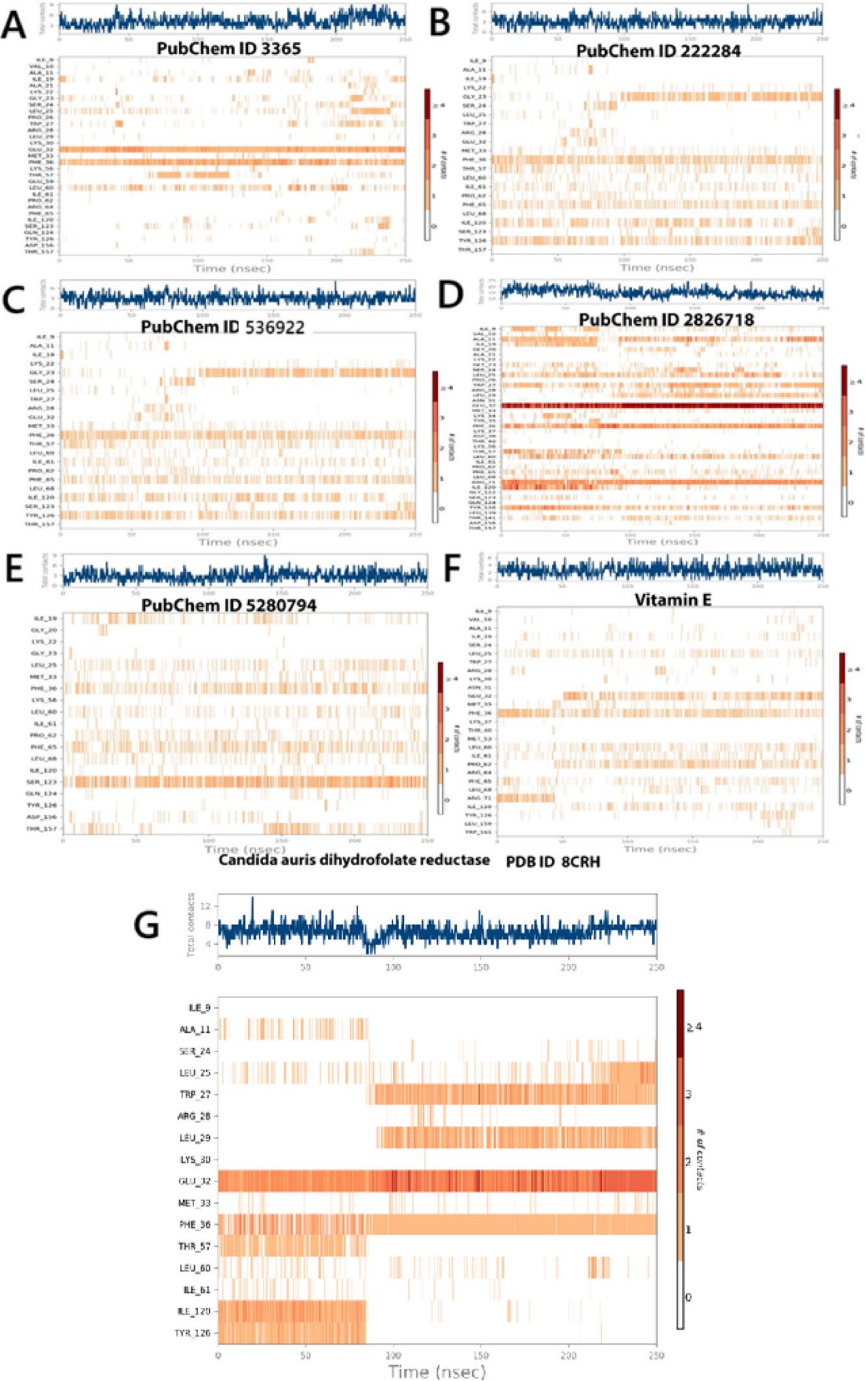
Ligand–protein contact plot for compounds 8CRH complexes during simulation trajectory. (**A**) Fluconazole, (**B**) β-sitosterol, (**C**) Dihydroxanthin, (**D**) Echinocandin, (**E**) Stigmasterol, (**F**) Vitamin E , and (**G**) crystal -form as a control.

Figure 10A–G depicts the contact frequency heatmaps for various ligands interacting with *Candida auris* dihydrofolate reductase. The analysis highlights unique interaction profiles for each ligand, with the persistence of contacts serving as a measure of binding stability. Fluconazole demonstrated frequent and sustained interactions with GLU32, PHE36, and ILE120, indicating stable binding characterized by hydrophobic and ionic interactions (Fig. 10A). β-sitosterol primarily engaged with ILE120 and PHE36, suggesting a deeply embedded hydrophobic binding mode with minimal polar interaction, consistent with its low polar surface area (Fig. 10B). Dihydroxanthin exhibited moderately distributed contacts across residues such as PHE36, TYR126, and ILE9, reflecting a transient yet balanced interaction pattern (Fig. 10C). Echinocandin displayed the most robust and consistent contact profile, maintaining multiple high-frequency interactions with GLU32, MET33, PHE36, TRP27, and ILE9. This pattern aligns with its superior MMGBSA binding energy and minimal conformational fluctuations, confirming its strong affinity and structural compatibility with the binding site (Fig. 10D). Stigmasterol showed stable yet slightly variable contacts with ILE120, GLU32, and PHE36, indicative of tight, predominantly hydrophobic binding (Fig. 10E). Vitamin E maintained moderate but consistent interactions with GLU32, MET33, and PHE36, supporting its low RMSD values and favorable binding energy (Fig. 10F). The co-crystallized ligand (control) exhibited intense and steady interactions, primarily involving GLU32, MET33, and PHE36, validating the simulation setup (Fig. 10G). Notably, both Echinocandin and Vitamin E demonstrated comparable or superior contact patterns relative to the control, underscoring their potential as strong DHFR binders in *C. auris*.

#### Residue cross-correlation dynamics of 8CRH–ligand complexes

**Fig. 11:**
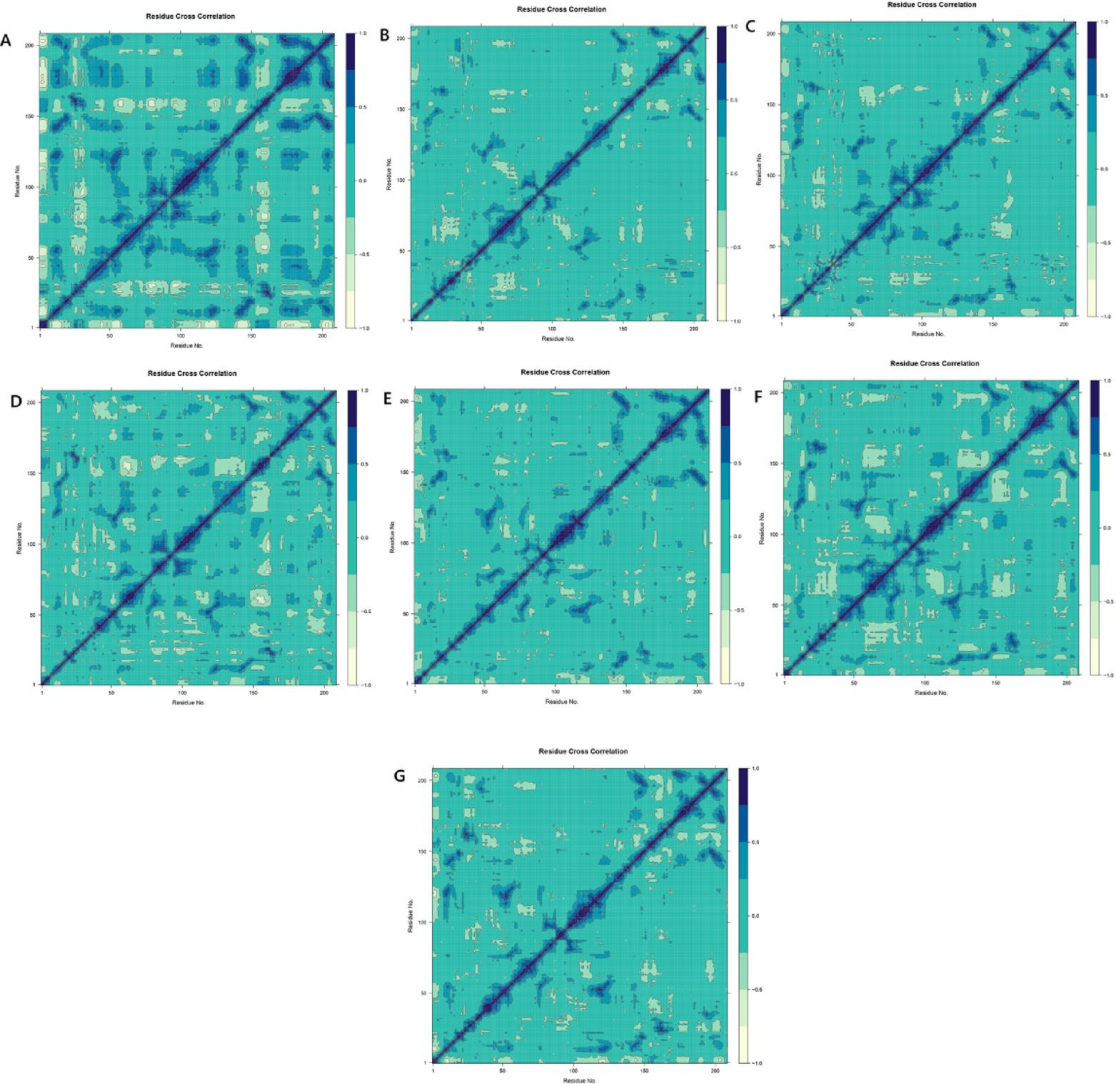
Residue Cross-Correlation Dynamics of 8CRH–Ligand Complexes: (**A**) Fluconazole, (**B**) β-sitosterol, (**C**) Dihydroxanthin, (**D**) Echinocandin, (**E**) Stigmasterol, (**F**) Vitamin E , and (**G**) crystal form as a control. Positive correlation (blue) denotes coordinated movement, negative correlation (pale yellow) indicates anti-correlated motion, and neutral correlation (green to cyan) indicates minimal or no directional correlation.

The dynamic cross-correlation matrix (DCCM) analysis was applied to explore the internal residue motion and coordination of *Candida auris* dihydrofolate reductase (8CRH) when complexed with various ligands over the course of 250 ns molecular dynamics simulations. These matrices elucidate the nature of correlated (positive), anti-correlated (negative), and uncorrelated residue motions, thereby illustrating the impact of ligand binding on protein dynamics. The 8CRH–fluconazole complex displayed moderate localized correlations with negligible global dynamic alterations, indicating stabilization of nearby residues (Fig. 11A). The β-sitosterol complex predominantly exhibited uncorrelated motions, suggesting minimal disruption to the protein’s overall dynamics, which aligns with its hydrophobic interaction characteristics (Fig. 11B). Dihydroxanthin induced moderate, distributed correlations, particularly in the central residue regions, indicating a balanced modulation of protein flexibility (Fig. 11C). Conversely, echinocandin prompted extensive correlated and anti-correlated motions, implying enhanced intra-protein communication and dynamic coordination, which supports its strong binding affinity (Fig. 11D). Stigmasterol, presented in Fig. 11E, local correlations with minor long-range effects, indicating a limited dynamic influence, whereas Vitamin E facilitated both strong local and moderate distal correlations, stabilizing intra-domain motions and flexible loops (Fig. 11F). The co-crystallized control ligand exhibited a balanced DCCM pattern, closely resembling those of Vitamin E and echinocandin, thereby highlighting their native-like interaction dynamics and potential as effective inhibitors (Fig. 11G).

#### The antimicrobial activity of *M. peregrina* and *M. oleifera’s* ethanol and aqueous extracts against *C. auris*, *C. albicans, and C. parapsilosis*

**Fig. 12:**
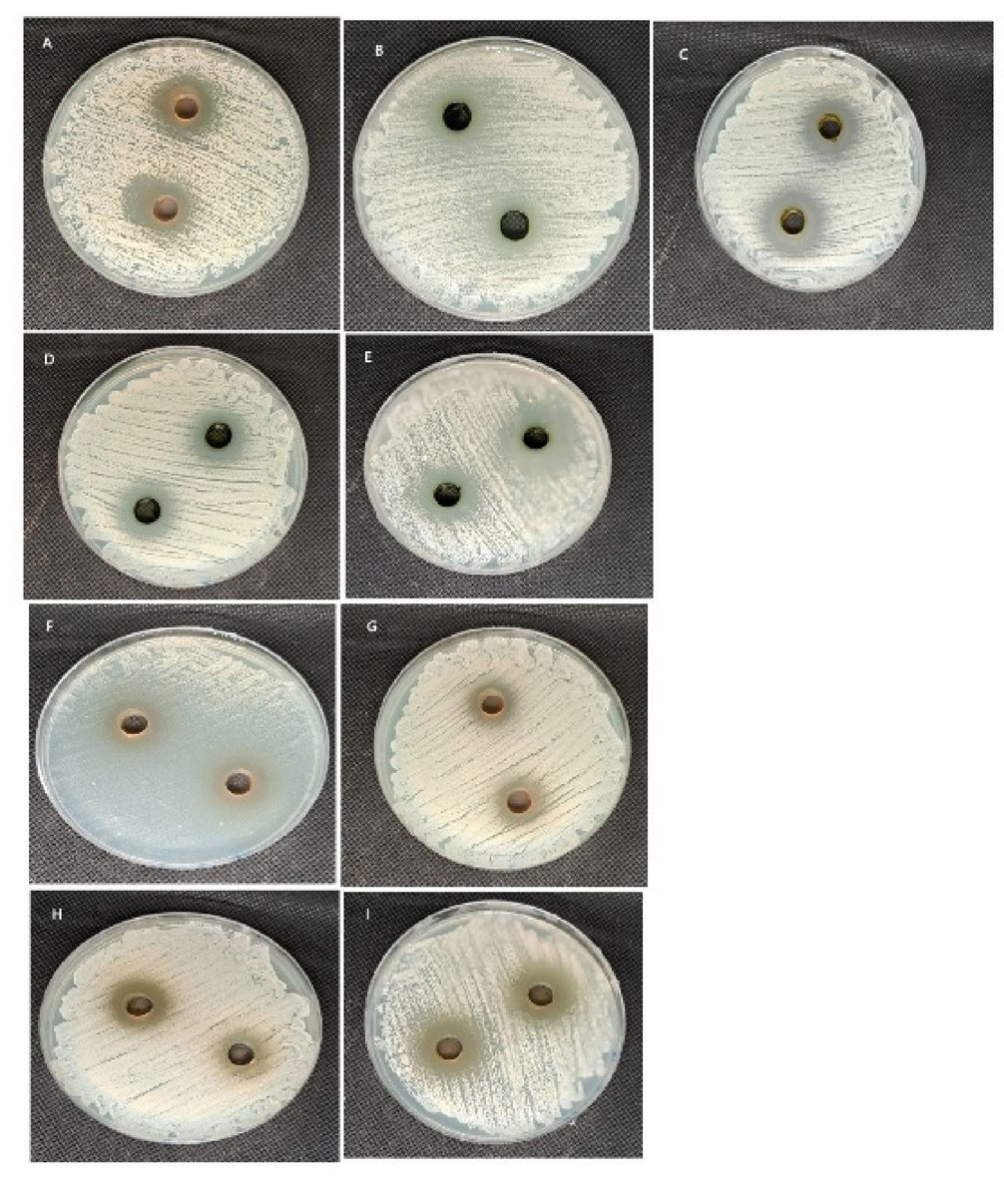
(**A**) The inhibition zone produced by the ethanol extract of *M. peregrina* against *C. auris* at a concentration of 7.5 mg/mL. (**B**) The inhibition zone produced by the ethanol extract of *M. oleifera* against *C. auris* at a concentration of 7.5 mg/mL. (**C**) The inhibition zone produced by the aqueous extract of *M. peregrina* against *C. auris* at a concentration of 7.5 mg/mL. (**D**) The inhibition zone produced by *M. oleifera* against *C. auris* at a concentration of 7.5 mg/mL.

**Fig. 13:**
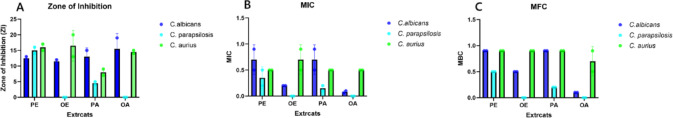
(**A**) Zone of inhibition (ZI). (**B**) MIC, and (**C**) MFC of Moringa plant extracts against all tested *Candida* strains at different concentrations in mg/ml. *M. peregrina* (Ethanol) ≡ (PE), *M. oleifera* (Ethanol) ≡ (OE), *M. peregrina* (Aqueous) ≡ (PA), *M . oleifera* (Aqueous) ≡ (OE).

Figure 12 presents the antifungal efficacy of four *Moringa* extracts against three *Candida* strains, benchmarked against standard antifungal agents. The table highlights significant variations in the ZI, MIC, and MFC across different extracts and fungal strains. A two-way ANOVA demonstrated a statistically significant interaction between extract type and organism for all parameters: ZI (*p* = 0.0009), MIC (*p* = 0.0364), and MFC (*p* = 0.0005), indicating that the efficacy of the extracts is significantly influenced by the *Candida spp*. The main effects of both extracts and organisms were also significant for all three parameters (*p* < 0.05), confirming that each factor independently affects antifungal response variations. Notably, the aqueous extract of *M. oleifera* showed the lowest MIC (0.08 mg/ml) and MFC (0.1 mg/ml) against *C. albicans*, indicating potent activity. Conversely, the aqueous and ethanol extracts of *M. oleifera* exhibited no inhibitory effect on *C. parapsilosis*, with ZI values of 0.0 mm, corresponding to high MICs (> 16 mg/ml). The standard antifungal susceptibility profile (as shown in the reference drug table) confirmed expected outcomes: *C. albicans* and *C. parapsilosis* were sensitive to all antifungals, whereas *C. auris* displayed multidrug resistance—with MIC values ≥ 256 µg/ml for fluconazole, ≥ 8 µg/ml for echinocandins (caspofungin and micafungin), and ≥ 64 µg/ml for flucytosine. This resistance pattern underscores the urgent need for alternative antifungal agents, such as plant-derived compounds, to combat emerging drug-resistant strains like *C. auris*. When *C. auris* was tested against *Moringa* extracts, it exhibited modest yet noteworthy susceptibility. The ethanol extracts of *M. peregrina* and *M. oleifera* showed the largest zones of inhibition (16 mm and 16.5 mm, respectively), indicating some inhibitory potential. However, the aqueous extracts demonstrated significantly weaker activity. For example, the aqueous extract of *M. oleifera* produced only a 4.5 mm ZI, while the *M. peregrina* aqueous extract showed 8 mm. These findings suggest that ethanol-extracted compounds may have greater bioactivity against *C. auris* than their aqueous counterparts. The MIC values further corroborate this pattern: The ethanol and aqueous extracts of *M. peregrina* had MICs of 0.5 mg/ml, while the ethanol extract of *M. oleifera* was slightly less effective (0.7 mg/ml). All MFC values for *C. auris* ranged from 0.7–0.9 mg/ml, indicating limited fungicidal activity. Although these concentrations are relatively high compared to typical antifungal agents, they remain noteworthy given *C. auris’s* resistance to conventional treatments (Fig. 13, Tables 4 and 5).

**Table 4:** MIC of the commercial antibiotics against all *Candida* strains.

Extracts	*C. albicans*	*C. parapsilosis*	*C. auris*
Organisms			
Fluconazole	< = 0.5	< = 0.5	> = 256
Voriconazole	< = 0.12	< = 012	> = 8
Caspofungin	0.12	0.25	> = 8
Micafungin	< = 0.06	0.5	> = 8
Amphotericin	0.5	< = 0.25	> = 2
Flucytosine	> = 0.64	< = 1	> = 64

**Table 5:** Zone of inhibition (ZI). (B) MIC and MFC of *Moringa* plant extracts against all *Candida* strains at different concentrations in mg/ml.

Extracts	Test	C. albicans	C. parapsilosis	C. auris
Organisms				
PE	ZI (mm)	12.5 ± 0.71	15 ± 1.41	16 ± 1.41
	MIC (mg/ml)	0.7 ±	0.35 ± 0.21	0.5 ± 0.0
	MFC (mg/ml)	0.9 ± 0.0	0.5 ± 0.0	0.9 ± 0.0
OE	ZI (mm)	11.5 ± 0.71	0.0 ± 0.0	16.5 ± 4.95
	MIC (mg/ml)	0.2 ± 0.0	> 16	0.7 ± 0.28
	MFC (mg/ml)	0.5 ± 0.0	> 16	0.9 ± 0.0
PA	ZI (mm)	13 ± 2.83	4.5 ± 0.71	8 ± 1.41
	MIC (mg/ml)	0.7 ± 0.28	0.15 ± 0.07	0.5 ± 0.0
	MFC (mg/ml)	0.9 ± 0.0	0.2 ± 0.0	0.9 ± 0.0
OA	ZI (mm)	15.5 ± 4.95	0.0 ± 0.0	4.5 ± 0.71
	MIC (mg/ml)	0.08 ± 0.02	> 16	0.5 ± 0.0
	MFC (mg/ml)	0.1 ± 0.0	> 16	0.7 ± 0.28

(ZI): Interaction *p* = 0.0009 (***). MIC: Interaction *p* = 0.0364 (*). MFC: Interaction *p* = 0.0005 (***). Significant interactions indicate that the effect of extracts varied across Candida strains. *M. peregrina* (Ethanol) ≡ (PE), *M. oleifera* (Ethanol) ≡ (OE), *M. peregrina* (Aqueous) ≡ (PA), *M . oleifera* (Aqueous) ≡ (OA).

## Discussion

The rise in multidrug-resistant fungal pathogens, notably *C. auris*, emphasizes the critical need for innovative antifungal approaches^16–18^. This study reveals that extracts from *M. peregrina and M. oleifera* exhibit notable antifungal properties against *C. auris*, *C. albicans*, and *C. parapsilosis*. The ethanolic extracts showed the most potent activity, which is consistent with ethanol’s ability to dissolve non-polar bioactive compounds such as sterols and terpenoids^14^. Our antifungal assays demonstrated inhibition zones reaching up to 16.5 mm and MIC values as low as 0.5 mg/mL for ethanolic extracts against *C. auris*. Although these concentrations are higher than those of standard antifungals, they are significant given *C. auris’s* high resistance to fluconazole (MIC ≥ 256 µg/mL), echinocandins (≥ 8 µg/mL), and flucytosine (≥ 64 µg/mL). These findings indicate that *Moringa* phytochemicals could form the basis for alternative or supplementary therapies^17,18^. These findings are consistent with previously published work highlighting the antifungal potential of *Moringa*-based preparations. For example, Yoshimatsu et al. (2023) demonstrated that *M. oleifera* leaf extract exerted strong antifungal effects against *C. albicans*, with MIC of 50 µg/mL and clear evidence of membrane damage and metabolic disruption^19^. Phytochemical analysis confirmed the presence of sterols (β-sitosterol, stigmasterol), vitamin E derivatives, and carotenoids like dihydroxanthin, and the FTIR spectra revealed prominent peaks corresponding to hydroxyl (O–H), carbonyl (C = O), and C–O functional groups, confirming the presence of alcohols, esters, phenols, and carboxylic acids, all these compounds and chemical groups known for their antimicrobial and antioxidant properties^14,20^. Similarly, our study found that the antifungal effect of *Moringa* extracts was more pronounced in ethanol preparations, supporting the view that ethanol efficiently extracts lipophilic antifungal compounds such as sterols and phenolics^21^.Vitamin E has been documented to enhance antibiotic effectiveness by disrupting microbial defense mechanisms^10,11^, while β-sitosterol disrupts bacterial membranes and is active against pathogens such as *Staphylococcus aureus*, *Escherichia coli*, and *Klebsiella pneumoniae*^11^. The presence of these compounds in our extracts reinforces the connection between *Moringa* phytochemistry and antifungal activity.

The results also align closely with the findings of the recent study investigating blended plant extracts of *Eremomastax speciosa*, *M. oleifera,* and *Senna alata*, which exhibited significant antifungal activity against *C. albicans, C. krusei,* and *C. parapsilosis* with MIC and MFC values of 125 and 250 µg/mL, respectively. The authors attributed this activity to polyphenols and flavonoids, particularly quercetin derivatives, which were identified as the major fungicidal constituents (M10 blend). These outcomes reinforce our observations that phytochemical constituents—especially polyphenols, sterols, and flavonoids—play a crucial role in antifungal action. Just as quercetin-rich extracts in the synergistic study exhibited fungicidal properties, our GC–MS analysis confirmed the presence of major bioactive metabolites such as β-sitosterol and stigmasterol, which are also known to contribute to membrane disruption and inhibition of fungal growth. Together, the agreement between our findings, the single-plant *Moringa* study by Yoshimatsu et al. (2023), and the multi-plant synergistic study suggests that *Moringa* derived phytochemicals have a reproducible antifungal effect across different *Candida spp.*even though resistance profiles differ^19^. Notably, while earlier studies focused on *C. albicans* and other non-resistant species, our work advances this field by demonstrating that *Moringa* extracts retain inhibitory effects even against multidrug-resistant *C. auris*. This indicates that the mechanism may extend beyond classical ergosterol interference and supports the therapeutic potential of plant-based metabolites as alternative antifungal agents.

A unique aspect of this research is the targeting of *C. auris* dihydrofolate reductase (DHFR, PDB ID: 8CRH) as a therapeutic target. Unlike conventional antifungal targets like ERG11 or FKS1, DHFR is essential for DNA synthesis and less susceptible to resistance mutations. Molecular docking and molecular dynamics (MD) simulations confirmed robust and stable binding of β-sitosterol, stigmasterol, and vitamin E to DHFR, with binding free energies (− 65 to − 86 kcal/mol) surpassing those of fluconazole (− 27 kcal/mol). These results suggest that *Moringa* phytochemicals may operate through novel mechanisms beyond ergosterol inhibition^22^. Cytotoxicity assays further support the therapeutic potential of these extracts, as ethanolic preparations exhibited low toxicity to mammalian cells at antifungal concentrations, aligning with previous reports of favorable safety profiles for *Moringa*-derived compounds^14,20^. This is a crucial finding, as many plant-derived antimicrobials are limited by their non-selective cytotoxicity. Overall, our findings confirm that *M. peregrina* and *M. oleifera* are promising sources of natural antifungal agents. Ethanolic extracts showed superior efficacy, correlating with their richer phytochemical content. The identification of DHFR-binding sterols and vitamin E derivatives provides mechanistic insights and novel candidates for antifungal drug development. However, several limitations persist. First, the crude extracts contain a mixture of compounds; bioassay-guided fractionation and purification are necessary to identify the most active molecules. Second, while in vitro and in silico results are promising, in vivo validation in animal models is needed to establish pharmacokinetics, toxicity, and efficacy. Finally, potential synergistic effects between *Moringa* phytochemicals and conventional antifungals should be investigated, as combination therapy may enhance efficacy and reduce resistance. To conclude that, this investigation provides new evidence that *Moringa*-derived phytocompounds, particularly β-sitosterol, stigmasterol, and vitamin E, exhibit antifungal activity against multidrug-resistant *C. auris*. Through targeting DHFR, these natural compounds may overcome common resistance pathways. Our results focus in *Moringa* extracts as valuable candidates for further investigation in antifungal drug discovery.

## Conclusion

This research highlights the significant antifungal properties of extracts from* M. oleifera* and *M. peregrina* against *C. auris*, a notoriously resistant fungal pathogen. The study found that ethanolic extracts were more effective in inhibiting *C. auris* than aqueous extracts, as evidenced by significant interaction effects in a two-way ANOVA. In contrast to commercial antifungal agents, *C. auris* demonstrated complete resistance to fluconazole, voriconazole, amphotericin B, echinocandins, and flucytosine, emphasizing its multidrug-resistant characteristics and the urgent need for innovative therapeutic options. Phytochemical analysis using FTIR and GC–MS identified bioactive functional groups and key metabolites, including β-sitosterol and stigmasterol. Molecular docking studies revealed that these compounds exhibit strong binding affinities to fungal DHFR, a nontraditional target with reduced susceptibility to mutation-driven resistance. The stability of these interactions was further confirmed through molecular dynamics simulations. Notably, MTT assays showed that the extracts had minimal cytotoxic effects on Vero cells, indicating their safety. Overall, these findings underscore the antifungal potential of *Moringa*-derived phytochemicals and propose DHFR as a promising molecular target. This study lays the groundwork for future drug development initiatives aimed at creating plant-based, low-toxicity antifungal agents to combat resistant fungal infections like those caused by *C. auris*.

## Data Availability

All data generated or analyzed during this study are included in this published article .
